# ResSAT: Enhancing Spatial Transcriptomics Prediction from H&E- Stained Histology Images with Interactive Spot Transformer

**DOI:** 10.21203/rs.3.rs-4707959/v1

**Published:** 2024-08-09

**Authors:** Anqi Liu, Yue Zhao, Hui Shen, Zhengming Ding, Hong-Wen Deng

**Affiliations:** Tulane University; Tulane University; Tulane University; Tulane University; Tulane University

**Keywords:** Spatial transcriptomics, Hematoxylin and eosin images, transformer

## Abstract

Spatial transcriptomics (ST) revolutionizes RNA quantification with high spatial resolution. Hematoxylin and eosin (H&E) images, the gold standard in medical diagnosis, offer insights into tissue structure, correlating with gene expression patterns. Current methods for predicting spatial gene expression from H&E images often overlook spatial relationships. We introduce ResSAT (Residual networks - Self-Attention Transformer), a framework generating spatially resolved gene expression profiles from H&E images by capturing tissue structures and using a self-attention transformer to enhance prediction.Benchmarking on 10× Visium datasets, ResSAT significantly outperformed existing methods, promising reduced ST profiling costs and rapid acquisition of numerous profiles.

## Background

The rapid advancement of spatial transcriptomics (ST) technology has revolutionized the field of RNA abundance quantification by offering remarkable spatial resolution for concurrent gene expression profiling and precise spatial spot localization ^1^. This breakthrough allows researchers to generate detailed maps of gene expression within tissues, providing unprecedented insights into cellular function and tissue organization ^2^. However, currently, the resource-intensive and time-consuming nature of ST profiling has limited its widespread application, creating a demand for more accessible methods.

In contrast, Hematoxylin and eosin (H&E) staining is a widely used histological technique that provides detailed insights into tissue structure and composition at a microscopic level ^3, 4^. H&E images are instrumental in medical diagnostics, offering clear visualizations of cellular morphology and tissue architecture ^5^. As the gold standard in many diagnostic procedures, H&E imaging is cost-effective, readily available, and extensively utilized in clinical settings.

Moreover, there is a close relationship between H&E staining and ST, as H&E images capture detailed cellular and tissue morphology that correlates with gene expression patterns ^6^. The staining highlights different cellular components: hematoxylin stains the cell nuclei blue, indicating areas of high DNA concentration, while eosin stains the cytoplasm and extracellular matrix pink, showing the structural context ^7^. These visual cues from H&E images reflect the underlying biological processes and molecular activities within the tissue ^3, 4^, providing a spatial map that can be linked to gene expression data. Previous studies have also demonstrated that changes in gene expression or genetic mutations often influence cell morphology, structure, and distribution, resulting in alterations in histological features ^8, 9^.

This correlation enables the use of H&E images to predict spatial gene expression profiles, leveraging the morphological context provided by the staining to infer molecular states. Recognizing the potential to integrate these two powerful tools, recent studies ^10–12^ have focused on developing computational approaches to predict ST data from H&E images. This innovative synergy aims to leverage the comprehensive tissue insights provided by H&E images to infer spatial gene expression profiles. By doing so, these approaches can potentially circumvent the limitations of ST technology, making high-resolution gene expression mapping more accessible and practical.

Several existing approaches, such as ST-Net ^6^, HisToGene ^11^, and BLEEP ^12^, have shown promising results in predicting expression from histology images. Both ST-Net and HisToGene treat expression prediction as regression tasks and train them in a feed-forward manner. ST-Net utilizes a ResNet50 image encoder, while HisToGene utilizes a vision transformer backbone. BLEEP, on the other hand, draws inspiration from contrastive language-image pretraining to establish a comparable joint embedding between spot expression profiles and their spatially paired image patches. Although HisToGene incorporates spatial location information, it does not explicitly consider spatial relationships between different locations.

It’s worth highlighting existing methods capable of generating spatially resolved expression predictions, either have limitations regarding the predicted panel (ST-Net, HisToGene, and BLEEP) or are prone to overfitting (HisToGene). Additionally, existing approaches in spatial expression prediction from H&E images often overlook the crucial relationships between different spatial locations, which provide essential context about tissue architecture, including the organization and interaction of cells, and spatial heterogeneity, and thus reflect variations in cellular composition and functions across different tissue regions. These factors may significantly impact biological interpretation and predictive accuracy ^13^. To address these challenges, we proposed a novel approach called ResSAT (Residual networks - Self-Attention Transformer) for predicting spatial transcriptomics profiles using H&E-stained histology images. We utilized a ResNet50 architecture to extract comprehensive image features from the H&E images, enabling our model to capture diverse characteristics of tissue structures and cellular compositions depicted in the images. Additionally, we introduced a self-attention transformer mechanism to identify and cluster spots with high correlation, allowing the model to focus on interactions between spots and enhance spatial gene expression prediction performance.

We validated the effectiveness of ResSAT by benchmarking its performance on two different mice brain datasets obtained from the 10× Visium platform. Our results demonstrated significant improvements over existing methods such as BLEEP ^12^, HisToGene ^11^, and ST-Net ^10^ in terms of mean correlations across all genes and mean correlations among the top 50 highly expressed genes.

This innovative approach significantly enhances the performance of spatial gene expression prediction from histology images. The proposed framework has the potential to substantially reduce the time and cost associated with spatial transcriptomics profiling, opening up new possibilities for acquiring numerous spatial transcriptomics profiles rapidly and reconstructing comprehensive 3D spatial transcriptomics from adjacent 2D spatial transcriptomics profiles.

## Results

### ResSAT enables gene expression prediction and consistently performs well.

To assess the predictive efficacy of ResSAT in quantifying gene expression, we applied our method to both the sagittal anterior (SA) and sagittal posterior (SP) datasets. We compared ResSAT to three other methods for spatial gene expression prediction, including BLEEP, HisToGene, and ST-Net. The predicted expression profiles from ResSAT showed the highest mean correlation with ground truth, achieving an increase in PCCs for both mean correlations of all genes (Table 1) and top 50 most highly expressed genes (HEGs) (Table 2) in the two different datasets. To ensure robustness and reliability, the evaluations were repeated five times, and the resulting mean and standard deviation values were computed for further analysis and validation.

Respectively, for each section within the SA and SP datasets, we trained ResSAT using one section and evaluated the correlation between predicted gene expression and actual gene expression on the other section. Specifically, after training on Section 2 and testing on Section 1 as shown in the Tables 1 and 2, we also trained on Section 1 and tested on Section 2 to validate the results. As illustrated in Fig. 2, ResSAT consistently yielded the highest PCCs between spatially resolved gene expression and actual gene expression across all sections in both datasets.

### Examining the effect of each module within ResSAT on the predicted gene expression results.

In order to better understand why ResSAT performs better than other methods, we conducted an analysis to see how each component contributes to its performance. We did this by removing certain modules of ResSAT and observing the impact on its ability to predict gene expression, as outlined in Figs. 3 and 4. We found that keeping all modules intact resulted in the strongest correlation between predicted and observed gene expression. Specifically, when we excluded the ResNet module and SAT module respectively, we noticed an average PCC decrease in performance in both the SA and SP datasets. This indicates that the ResNet module and SAT module are both crucial for ResSAT to effectively uncover the relationships between different spots. In summary, our ablation experiment highlights the importance of maintaining all modules within ResSAT to achieve optimal performance in predicting gene expression.

### ResSAT accurately predicts brain-related genes.

We examined whether the predicted gene expression by ResSAT accurately mirrors the actual expression of brain-related genes.

Within the SA dataset, we assessed the correlation between observed and predicted gene expression, computing the PCC for each gene. We then ranked these genes in descending order of their PCCs and selected the predicted genes with the top 5 highest correlations obtained from our method for visualization (*CALB2, GNG4, CDHR1, DOC2G*, and *SHISA8*), as shown in Fig. 5. *CALB2* (Calretinin) expression in the mouse olfactory bulb is associated with inhibitory interneurons ^32^. These interneurons play essential roles in regulating neural circuits involved in processing sensory information related to odors. *GNG4* (G Protein Subunit Gamma 4) is a gene encoding a subunit of heterotrimeric G proteins, which are involved in signal transduction pathways in neurons ^33^. In the mouse olfactory bulb, G proteins play a crucial role in mediating signaling pathways involved in odorant detection and processing.

Heterotrimeric G proteins, consisting of alpha, beta, and gamma subunits, are involved in transducing signals from odorant receptors to downstream effector molecules, leading to neuronal activation and olfactory perception ^33^. *CDHR1* (Cadherin-Related Family Member 1) is a gene expressed in the olfactory bulb of mice, where it likely contributes to the organization and maintenance of the olfactory sensory epithelium ^34, 35^. *DOC2G*, also known as Double C2 Domain Gamma, is a gene encoding a calcium-binding protein involved in vesicle exocytosis and neurotransmitter release. In the mammalian olfactory system, complex information processing starts in the olfactory bulb, whose output is conveyed by mitral cells (MCs) and tufted cells (TCs) ^36^. *DOC2G* was identified to be differentially expressed between MCs and TCs of the mouse ^36^. SHISA8, a member of the Shisa family of transmembrane proteins, has a broad role in synaptic function and neuronal development, suggesting its potential involvement in olfactory processing ^37^.

To further validate the robustness of ResSAT in predicting brain-related genes, we extended our analysis to the SP dataset, achieving similarly accurate predictions, as shown in Fig. 6. ResSAT enabled accurate prediction of key genes associated with mouse brain. Figure 6 shows the top 5 genes (*NRGN, CTXN1, PCP2, NNAT*, and *CAMK2A*) in the SA dataset predicted by ResSAT. NRGN (Neurogranin) is a gene encoding a protein found primarily in the brain, specifically in dendritic spines of neurons. It is involved in regulating synaptic plasticity and learning processes by modulating the function of calmodulin, a calcium-binding protein. *NRGN* has been implicated in various neurological disorders, including Alzheimer’s disease and schizophrenia ^38^. *CTXN1* (Cortexin-1) is a gene encoding a protein involved in neuronal development and synaptic function, especially highly expressed in cerebral cortex ^39^. *PCP2* (Purkinje Cell Protein 2) is a gene expressed primarily in Purkinje cells of the cerebellum ^40^. It encodes a protein involved in dendritic development, synaptic transmission, and calcium signaling within Purkinje cells. Mutations in *PCP2* have been linked to certain neurodevelopmental disorders. *NNAT* (Neuronatin) is a gene encoding a protein expressed in the brain, particularly in neurons, where it regulates neuronal development and function. It is involved in processes such as neuronal differentiation, synaptogenesis, and neurotransmitter release ^41^. *NNAT* has been implicated in neurological disorders and metabolic regulation ^42^. *CAMK2A* (Calcium/Calmodulin-Dependent Protein Kinase II Alpha) is a gene encoding a protein kinase involved in calcium signaling and synaptic plasticity. It plays a crucial role in neuronal excitability, synaptic transmission, and learning and memory processes. Dysregulation of *CAMK2A* has been implicated in various neurological disorders, including Alzheimer’s disease and epilepsy ^43^. These genes are known for their critical roles in neuronal function and brain development, reinforcing the model’s capability across different brain regions and datasets. This consistency underscores ResSAT’s reliability in predicting key genetic markers relevant to mouse brains.

## Discussion

To address the challenges inherent in spatial transcriptomics prediction, we devised a novel approach called ResSAT. Leveraging a ResNet50 architecture, we extracted comprehensive image features from provided H&E images. This method enabled our model to capture a wide range of tissue structures and cellular compositions depicted in the images. We then introduced a self-attention transformer mechanism to cluster spots exhibiting high correlation. This empowered the model to focus on interactions between spots, thereby enhancing spatial gene expression prediction performance.

In our experimental evaluations on benchmark ST datasets, our proposed method demonstrated its effectiveness in accurately predicting spatial gene expression patterns in two different mice brain datasets. We achieved significantly higher mean correlations across all genes and the top 50 most highly expressed genes (HEGs), representing substantial improvements compared to existing methods. Additionally, ResSAT exhibited similar expression patterns for the top 5 predicted genes compared to observed expression profiles. The results accurately predicted spatial correlations of genes, with the locations of predicted genes closely matching the spatial locations of observed genes. This underscores the efficacy of our approach in spatial transcriptomics prediction from H&E images, indicating its potential to generate numerous spatial transcriptomics profiles efficiently.

Our focus in this paper primarily centers on mouse brain datasets, laying the groundwork for our subsequent 3D reconstructed map of brain regions in spatial transcriptomics. Despite the successful prediction of specific genes showcased in Figs. 5 and 6, we acknowledge that the overall absolute correlations in Tables 1 and 2 remain low, indicating the challenging nature of the prediction task for the majority of genes. The low scores may stem from various factors, including the weak correlation between the expression of certain genes and morphological features, limitations in the detection of certain genes by the Visium platform resulting in less predictable expression, and the presence of experimental artifacts introducing non-biological variability into the data, independent of the image. Due to the relatively small number of tissue sections available, neither ResSAT nor other existing methods can reliably predict gene expression with high accuracy. However, ResSAT still demonstrates superior prediction accuracy compared to other methods. While the reliance on a relatively large training set poses a potential limitation for deep learning-based models, we anticipate that as more training ST data become available in the near future, ResSAT’s performance and robustness can be further enhanced.

## Conclusion

We introduced ResSAT, a novel framework for predicting spatial gene expression profiles from H&E-tained histology images using a ResNet50 architecture and a self-attention transformer mechanism. ResSAT effectively captures tissue structures and clusters correlated spots to enhance prediction performance. Our evaluations on mouse brain datasets demonstrated significant improvements over existing methods, with higher mean correlations and accurate spatial predictions.

Despite challenges like low absolute correlations for certain genes and limited tissue sections, ResSAT outperformed current methods, showing potential for efficient and cost-effective ST profiling. Future availability of more training data is expected to further enhance ResSAT’s performance and robustness, advancing the field of spatial transcriptomics.

## Methods

### Datasets: Two mouse brain datasets (SA and SP).

ST profiling of the anterior part and posterior part of the mouse brain tissue sagittal sections was generated with the Visium technology from 10× Genomics ^14–17^. Both datasets consist of serial H&E histology images and paired gene expressions at the spatial spots and their coordinates. The analyzed gene expression count matrices are outputs of the SpaceRanger pipeline ^18^.

The anterior sagittal dataset comprises two sets of H&E-stained histology images, alongside corresponding spatial gene expression data ^14, 15^. In slice 1, the spatial transcriptomics (ST) data encapsulates the expression profiles of 31,053 genes across 2,825 spots, given by read counts. Slice 2 features ST data for 31,053 genes across 2,696 spots, also detailed through read counts.

The posterior sagittal dataset includes two collections of H&E-stained histology images and their associated spatial gene expression data ^16, 17^. For the first slice, spatial transcriptomics (ST) analysis reveals the expression levels of 32,285 genes across 3,355 spots, given by read count data. The second slice presents ST information for the same number of genes, but across 3,289 spots, with expression quantified similarly through read counts.

## Data preprocessing

For the H&E histology images, we extracted patches based on the size and location of each spot. Each patch was a 224×224 image centered around a spot, approximately 55μm on each side, and paired with the spot’s corresponding gene expression profile. For the spatial gene expression profiles of each tissue section, each spot was normalized to the total count and log normalized. The union of the top 1,000 most highly variable genes from each of the slices was used for training and prediction. Finally, the expression data of these samples were batch corrected using Harmony ^19^ before one of the slices was randomly selected to be held out for testing. For these two datasets, the slice 2 was selected to be held out for training, while the slice 1 was selected to be held out for testing.

### Learning image embedding for expression prediction.

Residual networks (ResNets) ^20^ are widely recognized architectures in image classification, initially acclaimed for their significant advancement upon introduction. They continue to be a benchmark in various image analyses ^21–23^ and serve as baselines in image studies introducing novel architectures ^24, 25^. Our focus in this paper was to extract highly representative features from H&E images using ResNet50. During the training phase, we utilized a dataset comprising N pairs of H&E images and their corresponding gene expressions. Each image patch was represented as a 3D tensor $X_{i}\in {\mathbb{R}}^{3\times W\times H}$, where N and H denoted the width and height of the patch, respectively. The associated gene expression $y_{i}$ was represented as a d-dimensional vector in ${\mathbb{R}}^{d}$.

Our objective was to develop a deep learning framework capable of accurately predicting gene expression from a given image patch. We conceptualized our model as comprising two main components: a backbone network, denoted as F(•), for initial feature extraction module, followed by a feature refinement module, G(•), to enhance the predictive capability of the extracted features. To optimize the performance of both modules, we employed the Mean Squared Error (MSE) loss function during the training process as follows:

$$L_{mse}=\sum_{i=1}^{N}\|y_{i}-G\left(F\left(X_{i}\right)\right){\|}_{l_{2}}$$

Where $l_{2}$ denotes the L-2 norm to measure the difference between predicted gene expression and ground-truth one.

### Spot-Interaction Discovery.

The emergence of transformers has led to their widespread application in image and omics data analysis, as demonstrated by numerous studies ^26–29^. Particularly, recent transformer-based architectures have drawn attention to self-attention and cross-attention mechanisms, offering means to capture interdependencies between different input modalities ^30, 31^.

In the context of our spatially resolved gene expression prediction approach, the Self-Attention Transformer (SAT) plays a crucial role in exploring spot-spot interactions. By clustering spots that exhibit high correlation, SAT enhances our model’s ability to represent gene expression accurately. This approach facilitates a deeper understanding of spatial relationships within the data, contributing to improved predictive performance.

**Figure 1:**
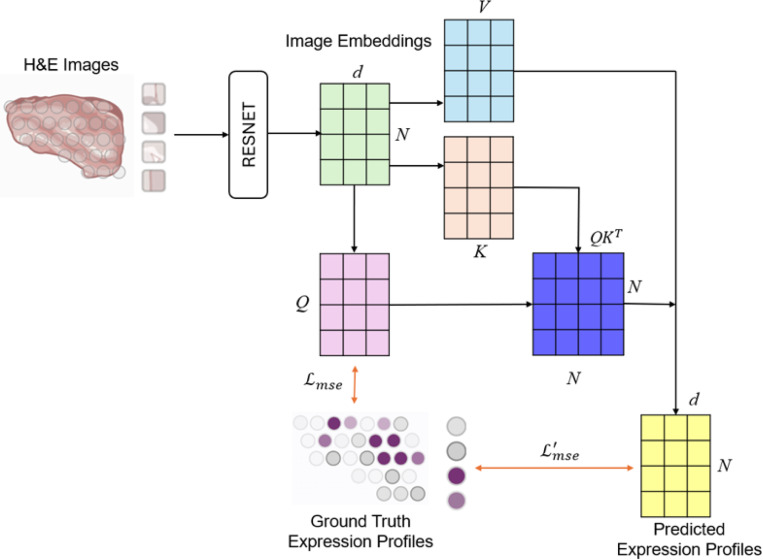
Overview of workflow.

**Figure 2:**
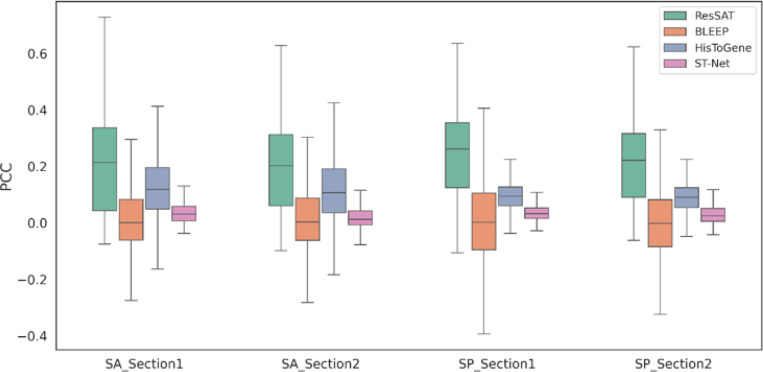
Comparative evaluation of spatial transcriptome prediction methods. SA_Section 1 represented Section 2 in the SA dataset was selected to be held out for training, while the Section 1 was selected to be held out for testing. SA_Section 2 represented Section 1 in the SA dataset was selected to be held out for training, while the Section 2 was selected to be held out for testing. SP_Section 1 represented Section 2 in the SP dataset was selected to be held out for training, while the Section 1 was selected to be held out for testing. SP_Section 2 represented Section 1 in the SP dataset was selected to be held out for training, while the Section 2 was selected to be held out for testing.

**Figure 3:**
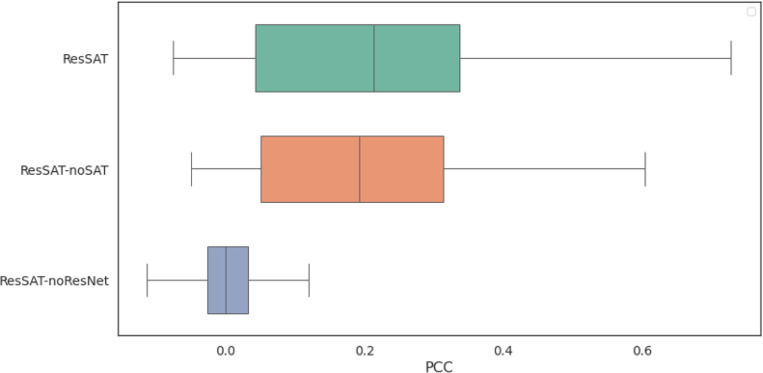
Ablation experiments on the SA dataset.

**Figure 4:**
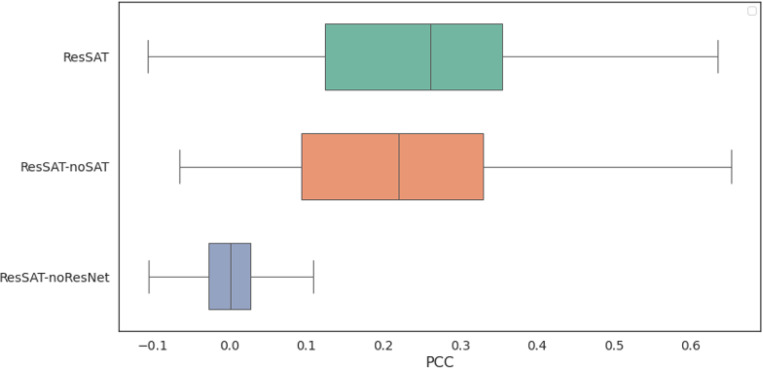
Ablation experiments on the SP dataset.

**Figure 5:**
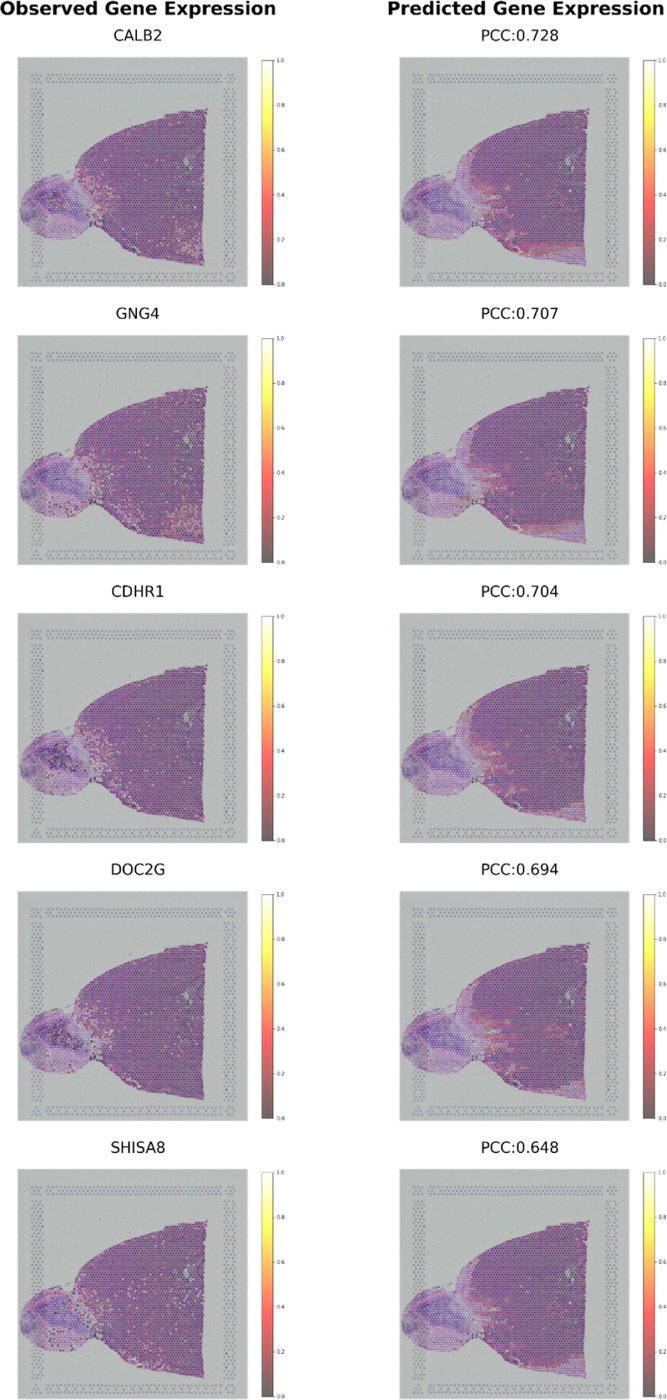
Visualization of the mouse brain (SA) dataset by the top five predicted genes with the highest correlations: *CALB2, GNG4, CDHR1, DOC2G*,and *SHISA8*.

**Figure 6:**
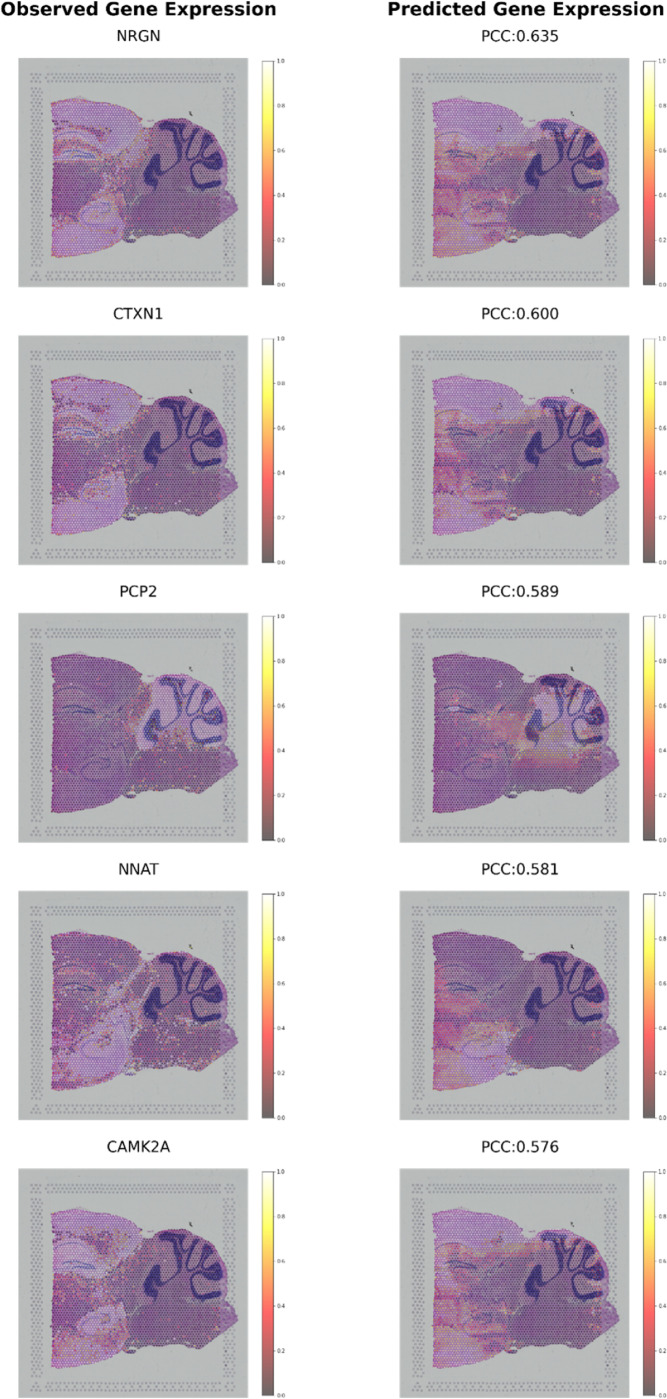
Visualization of the mouse brain (SP) dataset by the top five predicted genes with the highest correlations: *NRGN, CTXN1, PCP2, NNAT*,and *CAMK2A*.

In our endeavor to analyze H&E images for spot interaction, we proposed the development of a spot-interaction module, as shown in Fig. 1. This module aimed to refine predictions of gene expression. To streamline the model and minimize the number of parameters, we introduced a non-parametric attentive module. The operation of this attention module was defined by the following Equation:

$$A(Q,K,V)=\text{softmax}\left(\frac{QK^{\text{T}}}{\sqrt{d}}\right)V$$

where K, Q, and V represent the key, query, and value matrices of a Transformer module. To model the spot interaction, we aimed to learn a compact gene expression correlation between the spots in the training set. Here, $X_{i}$ represented the input features, and F(•) and G(•) denoted transformation functions. The aim of this formulation was to explore the matrix of spot-spot interactions. By doing so, it clustered spots that exhibit high correlation, thereby enhancing the robustness of the gene expression representation. Moreover, this approach elucidated the correlation among spots, improving the model’s inference capabilities. To enhance the model’s learning process by integrating knowledge of ground-truth gene expressions, we introduced a secondary MSE loss function. This additional MSE loss was formulated as follows:

$$L_{mse}^{\prime }=\sum_{i=1}^{N}\|y_{i}-A\left(G\left(F\left(X_{i}\right)\right)\right){\|}_{l_{2}}$$

Integrating dual objectives into a unified framework, we defined the overall loss function as follows:

$$L=L_{mse}+L_{mse}^{\prime }$$

This combined loss framework was designed to enhance the model’s predictive performance by balancing the feature extraction and interactive feature refinement. By carefully summing up the contribution of the primary and secondary MSE losses, the model was steered to pay detailed attention to the subtleties and complexities of gene expression data. This, in turn, was expected to improve the model’s capacity for capturing the intricate biological relationships that were represented within H&E images.

## Evaluated Metrics

In this study, we employed the Pearson correlation coefficient (PCC) to measure the spatial gene expression predicted by ResSAT with the observed gene expression, in order to assess their level of correlation. The PCC had a range of values between − 1 and 1. It was determined by dividing the covariance of two variables by the product of their individual standard deviations:

$$\text{PCC}=\frac{\mathrm{Cov}\left({\text{y}}_{\text{true}},\;{\text{y}}_{\text{pred}}\right)}{\sigma \;\left({\text{y}}_{\text{true}}\right)\;•\;\sigma \;\left({\text{y}}_{\text{pred}}\right)}$$

Where Cov(•,•) denotes covariance; ${\text{y}}_{\text{true}}$ and ${\text{y}}_{\text{pred}}$ represents the original gene expression and the gene expression obtained by prediction, respectively. σ(•) means standard deviation.

We computed the mean correlation of all genes as follows:

$$\bar{R}=\frac{1}{k}\sum_{i=1}^{k}\text{Corr}\left(y_{\text{true}}^{i},y_{\text{pred}}^{i}\right)$$

where $\text{Corr}\left(y_{\text{true}}^{i},y_{\text{pred}}^{i}\right)$ represented the Pearson correlation coefficient between the true and predicted expression values of the i-th gene, $y_{\text{true}}^{i}$ and $y_{\text{pred}}^{i}$, respectively. k is the number of all genes.

In addition to the mean correlation of all genes, we computed the mean correlation of the top 50 most highly expressed genes (HEGs) in predicted gene expression, compared with the observed gene expression. This metric provided insight into the performance of the prediction method specifically for genes that are highly expressed in the spatial context. Technically, the mean correlation of the top k′ HEGs was calculated as follows:

$${\bar{R}}_{50}=\frac{1}{k}\sum_{i=1}^{k}\text{Corr}\left(y_{\text{true}}^{i},y_{\text{pred}}^{i}\right)$$

Where $\text{Corr}\left(y_{\text{true}}^{i},y_{\text{pred}}^{i}\right)$ represented the Pearson correlation coefficient between the true and predicted expression values of the i-th gene, $y_{\text{true}}^{i}$ and $y_{\text{pred}}^{i}$, respectively. We set k′=50 in our experiments by default.

**Algorithm 1:** Spatial transcriptomics prediction using H&E-stained images.

**Require:** Image patch $\left(X\in {\mathbb{R}}^{N\times (3\times W\times H)}\right)$
Paired gene expression profile $\left(y_{\mathrm{true}}\in {\mathbb{R}}^{N\times d}\right)$

**Ensure: Expression prediction** $\left(y_{\text{pred}}\in {\mathbb{R}}^{N\times d}\right)$

1: **function** ResSAT (X)
2: $f_{x}\leftarrow G(F(X))N$ image embeddings
3: Q, K, V, $d\leftarrow f_{x}$ dimension of K as d
4: $y_{pred}\leftarrow \text{softmax}\left(QK^{T}/\bar{\sqrt{d}}\right)•V$ self-attention transformer
5: $L=L_{mse}+L_{mse}^{\prime }$
6: **return** $y_{\mathrm{true}}$, $y_{\mathrm{pred}}$, L

## Figures and Tables

**Table 1:** Mean correlations of all genes in predicted expression compared to ground truth expressions on held out datasets (Section 1).

Methods	SA	SP
ResSAT	0.2218 ± 0.0055	0.2437 ± 0.0026
BLEEP	0.0115 ± 0.0038	0.0091 ± 0.0054
HistoGene	0.1216 ± 0.0020	0.1215 ± 0.0016
ST-Net	0.0239 ± 0.0032	0.0340 ± 0.0061

Note: mean ± standard deviation

**Table 2:** Mean correlations of top 50 most highly expressed genes (HEGs) in predicted expression compared to ground truth expressions on held out datasets (Section 1).

Methods	SA	SP
ResSAT	0.3485 ± 0.0039	0.4003 ± 0.0252
BLEEP	0.0297 ± 0.0249	−0.0306 ± 0.0193
HistoGene	−0.0506 ± 0.0351	−0.0174 ± 0.04469
ST-Net	0.0774 ± 0.0043	0.0611 ± 0.0083

Note: mean ± standard deviation

## Data Availability

The source code of this work can be accessed at: https://github.com/Wonderangela/ResSAT under the GitHub. The raw public datasets analyzed during the current study are available in the 10X Genomics, https://www.10xgenomics.com/.

## References

[1] Jin Y, Zuo Y, Li G, Liu W, Pan Y, Fan T, Fu X, Yao X, Peng Y. Advances in spatial transcriptomics and its applications in cancer research. Mol Cancer. 2024;23(1):129. Epub 20240620. doi: 10.1186/s12943-024-02040-9. 38902727 PMC11188176

[2] Du J, Yang YC, An ZJ, Zhang MH, Fu XH, Huang ZF, Yuan Y, Hou J. Advances in spatial transcriptomics and related data analysis strategies. J Transl Med. 2023;21(1):330. Epub 20230518. doi: 10.1186/s12967-023-04150-2. 37202762 PMC10193345

[3] Titford M. The long history of hematoxylin. Biotech Histochem. 2005;80(2):73–8. doi: 10.1080/10520290500138372. 16195172

[4] Wittekind D. Traditional staining for routine diagnostic pathology including the role of tannic acid. 1. Value and limitations of the hematoxylin-eosin stain. Biotech Histochem. 2003;78(5):261–70. doi: 10.1080/10520290310001633725. 14989644

[5] Rosai J. Why microscopy will remain a cornerstone of surgical pathology. Lab Invest. 2007;87(5):403–8. Epub 20070402. doi: 10.1038/labinvest.3700551. 17401434

[6] Chen X, Lin J, Wang Y, Zhang W, Xie W, Zheng Z, Wong KC. HE2Gene: image-to-RNA translation via multi-task learning for spatial transcriptomics data. Bioinformatics. 2024;40(6). doi: 10.1093/bioinformatics/btae343. PMC11164830 38837395

[7] Chan JK. The wonderful colors of the hematoxylin-eosin stain in diagnostic surgical pathology. Int J Surg Pathol. 2014;22(1):12–32. Epub 20140109. doi: 10.1177/1066896913517939. 24406626

[8] McGranahan N, Swanton C. Clonal Heterogeneity and Tumor Evolution: Past, Present, and the Future. Cell. 2017;168(4):613–28. doi: 10.1016/j.cell.2017.01.018. 28187284

[9] Rizvi NA, Hellmann MD, Snyder A, Kvistborg P, Makarov V, Havel JJ, Lee W, Yuan J, Wong P, Ho TS, Miller ML, Rekhtman N, Moreira AL, Ibrahim F, Bruggeman C, Gasmi B, Zappasodi R, Maeda Y, Sander C, Garon EB, Merghoub T, Wolchok JD, Schumacher TN, Chan TA. Cancer immunology. Mutational landscape determines sensitivity to PD-1 blockade in non-small cell lung cancer. Science. 2015;348(6230):124–8. Epub 20150312. doi: 10.1126/science.aaa1348. 25765070 PMC4993154

[10] He B, Bergenstrahle L, Stenbeck L, Abid A, Andersson A, Borg A, Maaskola J, Lundeberg J, Zou J. Integrating spatial gene expression and breast tumour morphology via deep learning. Nat Biomed Eng. 2020;4(8):827–34. Epub 20200622. doi: 10.1038/s41551-020-0578-x. 32572199

[11] Pang M, Su K, Li M. Leveraging information in spatial transcriptomics to predict super-resolution gene expression from histology images in tumors. BioRxiv2021.

[12] Xie R, Pang K, Chung S, Perciani C, MacParland S, Wang B, Bader G. Spatially resolved gene expression prediction from histology images via bi-modal contrastive learning. Advances in Neural Information Processing Systems2024.

[13] Tanevski J, Flores ROR, Gabor A, Schapiro D, Saez-Rodriguez J. Explainable multiview framework for dissecting spatial relationships from highly multiplexed data. Genome Biol. 2022;23(1):97. Epub 20220414. doi: 10.1186/s13059-022-02663-5. 35422018 PMC9011939

[14] Mouse brain serial section 1 (sagittal-anterior). Available from: https://www.10xgenomics.com/datasets/mouse-brain-serial-section-1-sagittal-anterior-1-standard-1-1-0.

[15] Mouse brain serial section 2 (sagittal-anterior). Available from: https://www.10xgenomics.com/datasets/mouse-brain-serial-section-2-sagittal-anterior-1-standard-1-1-0.

[16] Mouse brain serial section 1 (sagittal-posterior). Available from: https://www.10xgenomics.com/datasets/mouse-brain-serial-section-1-sagittal-posterior-1-standard-1-0-0.

[17] Mouse brain serial section 2 (sagittal-posterior). Available from: https://www.10xgenomics.com/datasets/mouse-brain-serial-section-2-sagittal-posterior-1-standard-1-0-0.

[18] What is space ranger? Available from: https://www.10xgenomics.com/support/software/space-ranger/2.1/getting-started/what-is-space-ranger.

[19] Korsunsky I, Millard N, Fan J, Slowikowski K, Zhang F, Wei K, Baglaenko Y, Brenner M, Loh PR, Raychaudhuri S. Fast, sensitive and accurate integration of single-cell data with Harmony. Nat Methods. 2019;16(12):1289–96. Epub 20191118. doi: 10.1038/s41592-019-0619-0. 31740819 PMC6884693

[20] He K, Zhang X, Ren S, Sun J. Deep residual learning for image recognition. In Proceedings of the IEEE conference on computer vision and pattern recognition. 2016:770–8.

[21] Cubuk ED, Zoph B, Shlens J, Le QV. Randaugment: Practical automated data augmentation with a reduced search space. In Proceedings of the IEEE/CVF conference on computer vision and pattern recognition workshops. 2020:702–3.

[22] Yun S, Han D, Oh SJ, Chun S, Choe J, Yoo Y. Cutmix: Regularization strategy to train strong classifiers with localizable features. Proceedings of the IEEE/CVF international conference on computer vision. 2019:6023–32.

[23] Wightman R, Touvron H, Jégou H. Resnet strikes back: An improved training procedure in timm. arXiv preprint2021.

[24] Xie S, Girshick R, Dollár P, Tu Z, He K. Aggregated residual transformations for deep neural networks. In Proceedings of the IEEE conference on computer vision and pattern recognition. 2017:1492–500.

[25] Zhang H, Wu C, Zhang Z, Resnest: Split-attention networks. In Proceedings of the IEEE/CVF conference on computer vision and pattern recognition. 2022:2736–46.

[26] Gong P, Cheng L, Zhang Z, Meng A, Li E, Chen J, Zhang L. Multi-omics integration method based on attention deep learning network for biomedical data classification. Comput Methods Programs Biomed. 2023;231:107377. Epub 20230127. doi: 10.1016/j.cmpb.2023.107377. 36739624

[27] Wang S, Wang S, Yang D, Li M, Qing Z, Su L, Zhang L. Handgcat: Occlusion-robust 3d hand mesh reconstruction from monocular images. In 2023 IEEE International Conference on Multimedia and Expo (ICME). 2023:2495–500.

[28] Hu R, Singh A. Unit: Multimodal multitask learning with a unified transformer. In Proceedings of the IEEE/CVF International Conference on Computer Vision. 2021:1439–49.

[29] Nguyen S, Ng B, Kaplan AD, Ray P. Attend and decode: 4d fmri task state decoding using attention models. In Machine Learning for Health. 2020:267–79.

[30] Long NHB. Step catformer: Spatial-temporal effective body-part cross attention transformer for skeleton-based action recognition. arXiv preprint 2023.

[31] Wang J, Liao N, Du X, Chen Q, Wei B. A semi-supervised approach for the integration of multi-omics data based on transformer multi-head self-attention mechanism and graph convolutional networks. BMC Genomics. 2024;25(1):86. Epub 20240122. doi: 10.1186/s12864-024-09985-7. 38254021 PMC10802018

[32] Maksimova MA, Cansler HL, Zuk KE, Torres JM, Roberts DJ, Meeks JP. Interneuron Functional Diversity in the Mouse Accessory Olfactory Bulb. eNeuro. 2019;6(4). Epub 20190813. doi: 10.1523/ENEURO.0058-19.2019. PMC6712203 31358509

[33] Bakalyar HA, Reed RR. Identification of a specialized adenylyl cyclase that may mediate odorant detection. Science. 1990;250(4986):1403–6. doi: 10.1126/science.2255909. 2255909

[34] Nakajima D, Nakayama M, Kikuno R, Hirosawa M, Nagase T, Ohara O. Identification of three novel non-classical cadherin genes through comprehensive analysis of large cDNAs. Brain Res Mol Brain Res. 2001;94(1–2):85–95. doi: 10.1016/s0169-328x(01)00218-2. 11597768

[35] Sharon D, Blackshaw S, Cepko CL, Dryja TP. Profile of the genes expressed in the human peripheral retina, macula, and retinal pigment epithelium determined through serial analysis of gene expression (SAGE). Proc Natl Acad Sci U S A. 2002;99(1):315–20. Epub 20011226. doi: 10.1073/pnas.012582799. 11756676 PMC117558

[36] Koldaeva A, Zhang C, Huang YP, Reinert JK, Mizuno S, Sugiyama F, Takahashi S, Soliman T, Matsunami H, Fukunaga I. Generation and Characterization of a Cell Type-Specific, Inducible Cre-Driver Line to Study Olfactory Processing. J Neurosci. 2021;41(30):6449–67. Epub 20210607. doi: 10.1523/JNEUROSCI.3076-20.2021. 34099512 PMC8318078

[37] Abdollahi Nejat M, Klaassen RV, Spijker S, Smit AB. Auxiliary subunits of the AMPA receptor: The Shisa family of proteins. Curr Opin Pharmacol. 2021;58:52–61. Epub 20210421. doi: 10.1016/j.coph.2021.03.001. 33892364

[38] Pak JH, Huang FL, Li J, Balschun D, Reymann KG, Chiang C, Westphal H, Huang KP. Involvement of neurogranin in the modulation of calcium/calmodulin-dependent protein kinase II, synaptic plasticity, and spatial learning: a study with knockout mice. Proc Natl Acad Sci U S A. 2000;97(21):11232–7. doi: 10.1073/pnas.210184697. 11016969 PMC17183

[39] Coulter PM 2nd, Bautista EA, Margulies JE, Watson JB. Identification of cortexin: a novel, neuron-specific, 82-residue membrane protein enriched in rodent cerebral cortex. J Neurochem.1993;61(2):756–9. doi: 10.1111/j.1471-4159.1993.tb02183.x. 8336151

[40] Guan J, Luo Y, Denker BM. Purkinje cell protein-2 (Pcp2) stimulates differentiation in PC12 cells by Gbetagamma-mediated activation of Ras and p38 MAPK. Biochem J. 2005;392(Pt 2):389–97. doi: 10.1042/BJ20042102. 15948714 PMC1316275

[41] Oyang EL, Davidson BC, Lee W, Poon MM. Functional characterization of the dendritically localized mRNA neuronatin in hippocampal neurons. PLoS One. 2011;6(9):e24879. Epub 20110914. doi: 10.1371/journal.pone.0024879. 21935485 PMC3173491

[42] Braun JL, Geromella MS, Hamstra SI, Fajardo VA. Neuronatin regulates whole-body metabolism: is thermogenesis involved? FASEB Bioadv. 2020;2(10):579–86. Epub 20200902. doi: 10.1096/fba.2020-00052. 33089074 PMC7566048

[43] Hudmon A, Schulman H. Structure-function of the multifunctional Ca2+/calmodulin-dependent protein kinase II. Biochem J. 2002;364(Pt 3):593–611. doi: 10.1042/BJ20020228. 11931644 PMC1222606

